# Supplementary material for: Improving mental ill-health with psycho-social group interventions in South Asia–A scoping review using a realist lens
Source: PLOS Glob Public Health. 2023 Aug 28;3(8):e0001736. doi: 10.1371/journal.pgph.0001736 (PMC10461838; doi:10.1371/journal.pgph.0001736)
Supplement: S1 File — (DOCX) [file pgph.0001736.s001.docx]

| **Interventions (completed studies)** | **Study** | **Age Range (y)** | **Number of children** | **Caste** | **Income / Poverty** | **Employment** | **Level of Education** | **Sex** |
| --- | --- | --- | --- | --- | --- | --- | --- | --- |
| **Widows in North East India**  Other study used same group | Kermode study(1) | Overall: 20-52 | Mean=2.4 | Not reported | Not reported | Employed= 16%; Unemployed=81%; Missing=3%. | None=9%;  Primary=8%;  Secondary=53%;  Tertiary=30%. | 100% female. |
| **Service users in Gujarat**, India | Pathare study (2) | 19 - >50 | Mean 2.3 | Not reported | Not reported | Employed=52%  Unemployed = 48% | Primary 39%;  Secondary 25%;  Tertiary 35% | 28% female |
| **People with mental distress in Uttarakhand, India** | Mathias study (3) | 14 – 60+ | Not reported | Dalit / Tribal 21.9%  Other backward castes 47.1%  General 31% | Poor (housing temporary materials) 38% | Employed = 40%  Unemployed/ student = 39.7% | Not reported | 60.6% female |
| **Participatory groups with mothers across South Asia** | Tripathy study (4) | 95% between 15-49 years | Not reported | • Intervention:  Scheduled tribe N=1849 (75%); scheduled caste N=80 (3%); other backward caste N=520 (21%)  • Control: Scheduled tribe N=1557 (70%); scheduled caste N=64 (3%); other backward caste N=606 (27%) | Not reported | Not reported | • Intervention: None N=1908 (78%); primary N=143 (6%); secondary or higher N=405 (16%); cannot read N=1906 (78%); can read N=550 (22%). • Control: None N=1533 (69%); primary N=125 (6%); secondary or higher N=577 (26%). Cannot read N=1566 (70%); can read N=669 (30%). | 100% female. |
|  | Clarke study (5) | Mean age 24.7  (baseline) | Mean 2.7 (baseline) | Not reported | 36.6% of participants | Not reported |  |  |
| **Women in self-help groups in India** | Rao study (6) | • 18-70 (2007)  • 20-65 (2011) | • Mean 3.0 | • Scheduled caste N=130  • Other N=160 | • Poor enough to need micocredit. | Not reported | Not reported. | 100% female. |
| **Women surviving tsunami in India** | Becker 2009 study (others did not report this detail) (7) | • Intervention: <26 (16%);  26-35 (33%); 36-45 (34%); 46-55 (15%); ≥56 (2%).  • Control:  <26 (18%);  26-35 (45%); 36-45 (25%); 46-55 (11%); ≥56 (1%) | Not reported | Not reported | • Intervention: <1000 Rupees/month (81%); 1001-3000 Rupees/month (16%); 3001-5000 (2%); >5000 (1%)  • Control: <1000 Rupees/month (85%); 1001-3000 (14%); 3001-5000 (1%); >5000 (0%) | Not reported. | • Intervention:  None (54%);  1-4 years (20%);  5-7 years (15%);  8-10 years (11%). • Control:  None (61%);  1-4 years (4%);  5-7 years (30%);  8-10 years (5%). | 100% female. |
| **Women in North India** | Multiple studies(8-11) | 16-65 | Not reported. | Not reported | Not reported. | Not reported. | None (29%); Primary (32%); Secondary (27%); Bachelors/Masters (12%). | 100% female |
| **Women with depression in Bangladesh** | Karasz 2021 (12) | 18 -40  Interv 26.0y  Ctrl 26.1y | 57.1% < 2  42.9% <2 | Not reported | I 35.1% debt  C 40.0% debt | Not reported | I Mean 6y completed educn  C Mean 4 y completed educn | 100% female |
| **Adults in Nepal** | Jordans study (13) | Not reported. Average age: intervention 36.5; control 34.9. | Not reported. | • Intervention: Brahmin/Chhetri (34%); Dalit (30%);  Janajati/Giri (36%). • Control: Brahmin/Chhetri (23%); Dalit (32%);  Janajati/Giri (45%). | Family income sufficient to sustain yourself? (mths per yr). • Intervention:  Always (9-12)=27%, Very much (6-9)=33%, A quite bit (3-6)=29%, A little (1-3)=10%  • Control:  Always=15%,  Very much=34%,  A quite bit=34%,  A little=17% | Not reported. | • Intervention:  None (38%);  primary (32%);  secondary (21%);  higher secondary (9%). • Control:  None (43%);  primary (34%);  secondary (19%);  higher secondary (4%). | • Interventn: Male 51%; Female 49%. • Control: Male 51%; Female 49%. |
| **Problem management Plus**  NB Other studies in this group were qualitative or protocols | Khan study (14) | Not reported | 2 or more children under 7 years old: intervention 29%; control 37%. | Not reported. | Not reported.  Financially empowered: intervention 46%; control 43%. | • Intervention: Paid work 17%; housekeeping 81%.  • Control: paid work 27%; housekeeping 68%. | • Intervention: None 70%; primary 15%; secondary 10%; higher 5%. • Control: None 68%; secondary 10%; higher 5%. | 100% female. |
|  | Rahman study(15) | Not reported. Average age: intervention 37.3; control 35.2. | Intervention: mean=4.8; Control: mean=4.6 | Not reported | Not reported. | • Intervention:  Housewife 88%;  Self-employed or paid work 10%;  non-paid work <2%;  • Control:  Housewife 81%; self-employed or paid work 13%;  non-paid work< 4%; | • Intervention: No schooling 81%; primary 9%; secondary 9%; college and university <1%; missing data 1%. |  |
|  | Sangraula study (16) | Mean age Interven 46.7;  control 49.3 | Not reported | Intervention/ control gp  Brahmin/Chetri 21/45%  Dalit 15/23%  Danuwar 38/5%  Other 26/26% | Self-perceived SES in intervention/ control  Bad or V bad 13/46%  Normal 62/ 45%  Good 23/8%  V good 2/0% | • Intervention  Farmer – 33%  Office – 3%  Business – 7%  Hwife – 48%  Daily wage/ other 11% | • Intervention  Illiterate 59%;Informal/primary 28%; Sec/higher sec 14%; University 0%  • Control  Illiterate 80%;Informal/primary 17%; Sec/higher sec 3%; University 0% | Intervention and control:  17% male  83% female |
| **Tsunami survivors in the Andaman Islands** | Telles study(17) | 28-50 | Not reported | Not reported | Not reported | Not reported. | Not reported. | Not reported. |
| **Social support among Pakistani women** | Hirani study (18) | Mean intervention 31; Mean control 32. | Not reported. | Not reported. | Intervention:  <10K Rs/mth (3%);  10-24K Rs/mth (67%);  25-40K Rs/mth (25%). • Control:  <10K Rs/mth (5%);  10-24K Rs/mth (50%);  25-40K Rs/mth (37%). | • Intervention: 22%. • Control: 20%. | • Intervention: Primary (8%); secondary (48%); higher (28%).  • Control: Primary (10%); secondary (40%); higher (27%). | 100% female. |
| **Economic skill building among Pakistani women** | Hirani studies (19-21) | 20-45 | Not reported | Not reported | Average household income US$55. | • ESB: 1 (11%). • Counselling: 3 (33%).  • Control: 1 (12%). | Unclear but majority reported <4 years education. | 100% female |
| **Adults with depressive symptoms in Pakistan** | Saleem study (22) | 18 – 65 (mean aged 39y) | Not reported | Not reported | Not reported | Not reported | Not reported | 23/30 participants female |
| **Thinking Healthy** (other studies were qualitative) | Sikander study (23) | 18-44 | Mean=2; range=1-4. | Not reported. | Not reported. | • Intervention:  N=20 (7%).  • Control:  N=16 (6%) | • Intervention: no formal education (N=52, 18%); primary (N=68, 24%); secondary (N=120, 42%); higher secondary (N=25, 9%); university or higher (N=18, 6%). • Control: no formal education (N=55, 19%); primary (N=71, 25%); secondary (N=113, 39%); higher secondary (N=21, 7%); university or higher (N=27, 9%). | • Interventn: N=20 (7%).  • Control: N=16 (6%). |
|  | Nusrat study(24) | 18-44 | Not reported | Not reported | Not reported | Not reported | Not reported | • Interventn: n=811 women (100%) |
|  | Maselko study (25) | 18 – 44  Mean 26.7 | Mean=2; range=1-4. | Not reported | Not reported | Not reported | As per Sikander study | • Interventn: N=20 (7%).  • Control: N=16 (6%). |

1. Kermode M, Devine A, Chandra P, Dzuvichu B, Gilbert T, Herrman H. Some peace of mind: assessing a pilot intervention to promote mental health among widows of injecting drug users in north-east India. BMC Public Health. 2008;8(1):294.

2. Pathare S, Funk M, Bold ND, Chauhan A, Kalha J, Krishnamoorthy S, et al. Systematic evaluation of the QualityRights programme in public mental health facilities in Gujarat, India. The British Journal of Psychiatry. 2021;218(4):196-203.

3. Mathias K, Corcoran D, Pillai P, Deshpande S, San Sebastian M. The effectiveness of a multi-pronged psycho-social intervention among people with mental health and epilepsy problems - a pre-post prospective cohort study set in North India. International Journal of Health Policy and Management. 2020.

4. Tripathy P, Nair N, Barnett S, Mahapatra R, Borghi J, Rath S, et al. Effect of a participatory intervention with women's groups on birth outcomes and maternal depression in Jharkhand and Orissa, India: a cluster-randomised controlled trial. Lancet. 2010;375(9721):1182-92.

5. Clarke K, Azad K, Kuddus A, Shaha S, Nahar T, Aumon BH, et al. Impact of a participatory intervention with women’s groups on psychological distress among mothers in rural Bangladesh: secondary analysis of a cluster-randomised controlled trial. PLoS One. 2014;9(10):e110697.

6. Rao K, Vanguri P, Premchander S. Community-based mental health intervention for underprivileged women in rural India: an experiential report. Int J Family Med. 2011;2011:621426.

7. Becker SM. Psychosocial care for women survivors of the tsunami disaster in India. Am J Public Health. 2009;99(4):654-8.

8. Gailits N, Mathias K, Nouvet E, Pillai P, Schwarz L. Women’s freedom of movement and participation in psychosocial support groups: a qualitative study in Northern India. BMC Public Health. 2019;19(725).

9. Mathias K, Mathias J, Goicolea I, Kermode M. Strengthening community mental health competence – a realist informed case study from Dehradun, North India. Health & Social Care in the Community. 2018;26(1):e-179-90.

10. Gailits N, Mathias K, Nouvet E, Pillai P, Schwarz L. Transformed women, transformed communities: Impact of mental health support groups for North Indian women. Annals of Global Health. 2017;83(1):94-109.

11. Gailits NS. “How can we share when we don't go out?" psychosocial support groups for North Indian women Canada: McMasters; 2017.

12. Karasz A, Anne S, Hamadani JD, Tofail F. The ASHA (Hope) Project: Testing an Integrated Depression Treatment and Economic Strengthening Intervention in Rural Bangladesh: A Pilot Randomized Controlled Trial. Int J Environ Res Public Health. 2021;18(1):279.

13. Jordans M, Aldridge L, Luitel N, Baingana F, Kohrt B. Evaluation of outcomes for psychosis and epilepsy treatment delivered by primary health care workers in Nepal: a cohort study. International journal of mental health systems. 2017;11(1):70.

14. Khan M, Hamdani S, Chiumento A, Dawson K, Bryant R, Sijbrandij M, et al. Evaluating feasibility and acceptability of a group WHO trans-diagnostic intervention for women with common mental disorders in rural Pakistan: a cluster randomised controlled feasibility trial. Epidemiology and psychiatric sciences. 2019;28(1):77-87.

15. Rahman A, Khan MN, Hamdani SU, Chiumento A, Akhtar P, Nazir H, et al. Effectiveness of a brief group psychological intervention for women in a post-conflict setting in Pakistan: a single-blind, cluster, randomised controlled trial. The Lancet. 2019;393(10182):1733-44.

16. Sangraula M, Turner EL, Luitel NP, van‘t Hof E, Shrestha P, Ghimire R, et al. Feasibility of Group Problem Management Plus (PM+) to improve mental health and functioning of adults in earthquake-affected communities in Nepal. Epidemiology and psychiatric sciences. 2020;29:e130.

17. Telles S, Naveen K, Dash M. Yoga reduces symptoms of distress in tsunami survivors in the Andaman Islands. Evid Based Complement Alternat Med. 2007;4(4):503-9.

18. Hirani SS, Norris CM, Van Vliet KJ, Van Zanten SV, Karmaliani R, Lasiuk G. Social support intervention to promote resilience and quality of life in women living in Karachi, Pakistan: a randomized controlled trial. International Journal of Public Health. 2018;63(6):693-702.

19. Hirani SS, Karmaliani R, McFarlane J, Asad N, Madhani F, Shehzad S, et al. Development of an economic skill building intervention to promote women's safety and child development in Karachi, Pakistan. Issues Ment Health Nurs. 2010;31(2):82-8.

20. Hirani SS, Karmaliani R, McFarlane J, Asad N, Madhani F, Shehzad S. Testing a community derived intervention to promote women’s health: preliminary results of a 3-arm randomized controlled trial in Karachi, Pakistan. South Online J Nurs Res. 2010;10(10).

21. Asad N, McFarlane J, Hirani S, Madhani F, Shehzad S. Applying community participatory methods to meet women's mental health needs in Karachi, Pakistan. Pak J Psychol. 2011;42(1):119.

22. Saleem S, Baig A, Sajun S, Bird V, Priebe S, Pasha A. A mixed methods exploration of the role of multi-family groups in community treatment of patients with depression and anxiety in Pakistan. International Journal of Mental Health Systems. 2021;15(1):1-8.

23. Sikander S, Ahmad I, Atif N, Zaidi A, Vanobberghen F, Weiss HA, et al. Delivering the Thinking Healthy Programme for perinatal depression through volunteer peers: a cluster randomised controlled trial in Pakistan. The Lancet Psychiatry. 2019;6(2):128-39.

24. Nusrat H, Fatima B, Kiran T, Husain M, Shah S, Hina F, et al., editors. Intervention for maternal depression and early child development: An RCT from urban slums of Pakistan. Eur Psychiatry; 2018: Elsevier.

25. Maselko J, Sikander S, Turner EL, Bates LM, Ahmad I, Atif N, et al. Effectiveness of a peer-delivered, psychosocial intervention on maternal depression and child development at 3 years postnatal: a cluster randomised trial in Pakistan. The Lancet Psychiatry. 2020;7(9):775-87.
