# Supplementary material for: Improving mental ill-health with psycho-social group interventions in South Asia–A scoping review using a realist lens
Source: PLOS Glob Public Health. 2023 Aug 28;3(8):e0001736. doi: 10.1371/journal.pgph.0001736 (PMC10461838; doi:10.1371/journal.pgph.0001736)
Supplement: S3 File — (DOCX) [file pgph.0001736.s003.docx]

Sample Medline Search

| 1. | (india or pakistan or "Sri Lanka" or Bangladesh or Afghanistan or Bhutan or Maldives).mp. [mp=ti, ab, hw, tn, ot, dm, mf, dv, kf, fx, dq, bt, id, cc, nm, ox, px, rx, ui, sy, tx, ct] |  |  |  |  |
| --- | --- | --- | --- | --- | --- |
| 2. | (("mental health" or "mental stress" or "mental disease" or "mental disorder" or "mental disorders") and ("psychosocial" or "task shifting" or "health care personnel" or "community care" or "social support" or "mental health care" or "group therapy")).mp. [mp=ti, ab, hw, tn, ot, dm, mf, dv, kf, fx, dq, bt, id, cc, nm, ox, px, rx, ui, sy, tx, ct] |  |  |  |  |
| 3. | 1 and 2 |  |  |  |  |

Sample MEDLINE search. The search did not rely on MeSH terms, given that many papers had not been indexed at the time of search.

Term 1: Countries

Term 2: Synonyms for mental health AND indicator terms for psychosocial interventions

Indicator terms for psychosocial interventions were gathered based on the initial mini-review
