# Supplementary material for: Improving mental ill-health with psycho-social group interventions in South Asia–A scoping review using a realist lens
Source: PLOS Glob Public Health. 2023 Aug 28;3(8):e0001736. doi: 10.1371/journal.pgph.0001736 (PMC10461838; doi:10.1371/journal.pgph.0001736)
Supplement: Attachment — Submitted filename: 2 Response to reviewers and editors Feb2023.docx [file pgph.0001736.s004.docx]

Journal Requirements:

1. Please amend your detailed Financial Disclosure statement. This is published with the article. It must therefore be completed in full sentences and contain the exact wording you wish to be published.
 
a. State the initials, alongside each funding source, of each author to receive each grant.
b. State what role the funders took in the study. If the funders had no role in your study, please state: “The funders had no role in study design, data collection and analysis, decision to publish, or preparation of the manuscript.”
c. If any authors received a salary from any of your funders, please state which authors and which funders.

If you did not receive any funding for this study, please simply state: “The authors received no specific funding for this work.”

*RESPONSE: Thanks, this has been amended accordingly*

2. Please provide separate figure files in .tif or .eps format only and remove any figures embedded in your manuscript file. Please also ensure that all files are under our size limit of 10MB.

For more information about figure files please see our guidelines:

<https://journals.plos.org/globalpublichealth/s/figures> 
<https://journals.plos.org/globalpublichealth/s/figures#loc-file-requirement>

*Response – we have provided TIF files*

3. Tables should not be uploaded as individual files. Please remove these files and include the Tables in your manuscript file as editable, cell-based objects. For more information about how to format tables, see our guidelines:

<https://journals.plos.org/globalpublichealth/s/tables>

*Response – tables are now inserted as per your guidelines. However we have found it challenging to left adjust Table 2 despite multiple efforts and I think Tables 2 and 4 would benefit from expert formatting through the journal.*

4. We have noticed that you have uploaded Supporting Information files, but you have not included a list of legends. Please add a full list of legends for your Supporting Information files after the references list.

Additional Editor Comments (if provided):

*Response - Legends for supporting information files now provided as the File titles*

[Note: HTML markup is below. Please do not edit.]

Reviewers' comments:

Reviewer's Responses to Questions

**Comments to the Author**

1. Does this manuscript meet PLOS Global Public Health’s [publication criteria](https://journals.plos.org/globalpublichealth/s/criteria-for-publication)? Is the manuscript technically sound, and do the data support the conclusions? The manuscript must describe methodologically and ethically rigorous research with conclusions that are appropriately drawn based on the data presented.

Reviewer #1: Yes

Reviewer #2: Partly

2. Has the statistical analysis been performed appropriately and rigorously?

Reviewer #1: N/A

Reviewer #2: I don't know

3. Have the authors made all data underlying the findings in their manuscript fully available (please refer to the Data Availability Statement at the start of the manuscript PDF file)?

The [PLOS Data policy](https://journals.plos.org/globalpublichealth/s/data-availability) requires authors to make all data underlying the findings described in their manuscript fully available without restriction, with rare exception. The data should be provided as part of the manuscript or its supporting information, or deposited to a public repository. For example, in addition to summary statistics, the data points behind means, medians and variance measures should be available. If there are restrictions on publicly sharing data—e.g. participant privacy or use of data from a third party—those must be specified.

Reviewer #1: Yes

Reviewer #2: No

4. Is the manuscript presented in an intelligible fashion and written in standard English?

PLOS Global Public Health does not copyedit accepted manuscripts, so the language in submitted articles must be clear, correct, and unambiguous. Any typographical or grammatical errors should be corrected at revision, so please note any specific errors here.

Reviewer #1: Yes

Reviewer #2: Yes

5. Review Comments to the Author

Please use the space provided to explain your answers to the questions above. You may also include additional comments for the author, including concerns about dual publication, research ethics, or publication ethics. (Please upload your review as an attachment if it exceeds 20,000 characters)

Reviewer #1: Thank you for the opportunity to review this paper. The paper aims to summarise what we know about interventions that target mentally ill adults at group-level, are delivered by community health workers and with a clearly defined psychosocial component.

The full title of the paper does not reflect the aforementioned summary well enough and could be ambiguous (…through(?) psycho-social (support?) groups or psycho-social group interventions(?)).

*Thanks for this comment – we have edited the title now to read as follows:*

*Improving mental ill-health with psycho-social group interventions in South Asia – a scoping review using a realist lens*

The link between initial search in 2019 and the inclusion of studies till February 2022 is confusing in the Abstract (clear in the paper).

*Thanks – we have edited the abstract to keep it clear.*

Authors assert that they use the realist approach for at least part of the study. Among the 3 questions that they outline on p.4, q3 is the realist component. The question in part is: “what enables these mechanisms to trigger positive outcomes?” In the realist view, mechanisms are the ones that trigger action (outcomes) within agents (in specific contexts). The current phrasing needs to be adapted to make it consistent with the the understanding of mechanisms in realist inquiry. Or else this inconsistency ought to be explained/discussed.

*Thanks for this comment, we have edited the questions to make them more consistent with a realist inquiry with the third question now phrased as:*

*(iii) What are possible mechanisms that trigger positive outcomes? What constrains positive outcomes?*

Among the mechanisms identified, the first one could benefit from a critical discussion among authors. I believe the framing of the mechanism ought to communicate the tendency within people that triggers action in some contexts (and not in others). The first mechanism appears to be the direct result/outcome of the actual mechanism. Please review. Compliments on the framing of the other mechanisms which appear very useful in program design.

*Thanks for the comment – we have discussed and re-phrased mechanism one as linking to a sense of belonging and connection – which then triggers participation.*

On p.13, reference is made to fig 1 which is supposed to be a middle range programme theory, but fig 1 is PRISMA on p.8. Authors possibly refer to fig 2.

*Thanks – we have changed the numbering*

There appears to be some conceptual confusion between middle-range theories & programme theories. There are instances when program theories can indeed be middle-range theories if they have sufficient degree of abstraction. However, what the authors attempt to do in figure 2 appears to be a comprehensive identification of intervention inputs/components, enablers, mechanisms and outcomes. This is NOT a middle-range theory nor can it be a good (initial/refined) programme theory. Programme theories are typically either explanations/schematics that describe what works, for whom, under what conditions and why. IN a sense they do leverage one or more outcome configurations and are an abstraction of one or more CMOs. But an aggregation of different aspects of diverse interventions found in literature into one schematic identified as a programme theory is problematic. And further conceptual confusion arises when the schematic (that is decontextualised!) is identified as “Middle-range programme theory”. Please carefully address this. To me this figure is an attempt at creating a comprehensive mapping of key enablers and mechanisms identified but NOT really either an MRT or a PT. Indeed, upon p.16, their assertions in “How do psychosocial groups lead to positive outcomes” are closer to explanatory MRTs that emerge from this review than this schematic in my assessment (albeit will need more work).

*Thanks for this comment – we have decided to focus on describing a program theory and not include a middle-range theory. The program theory we have phrased narratively as follows:*

In a context where people with psychosocial disability are socially excluded and have limited autonomy, psychosocial support groups can provide a safe space that facilitates a sense of mutual trust and the opportunity to participate in trusted relationship with peers. These relationships trigger a sense of inclusion and mutual social support, feeling able to manage one’s mental distress, belonging and collective strength. This triggers group members to take collective action that addresses structural and intermediary determinants of health and leads to outcomes of improved mental health and greater social participation and inclusion. We note that the distinction between outcomes and mechanisms and lines of causation are blurred and often bi-directional. A simplistic schematic summary of the context, enablers, mechanisms and outcomes is presented in Figure Two.

*We had also reviewed and edited Figure 2 and now name it as a schematic summary of key enablers, mechanisms and outcomes, recognising it is not sufficiently abstracted to be a middle range theory. We recognise that this diagram necessarily presents an over-simplified representation and we are very open to removing it altogether, or consider further edits (eg removing the element called ‘Enablers’) if Reviewer 1 believes this Figure is not helpful in this format.*

*We have pasted this Figure below for reviewer convenience:*

An important gap has been the absence of evidence/insights on key groups that are known to face social exclusion including (but not limited to) caste, religious minorities, LGBTQI, Adivasis/poor migrants/homeless. Without evidence on these, the discussion that the authors bring up on p.14 on “groups are socially acceptable” will need critical re-assessment.

*Thank you. We note that one of the interventions identified through our search terms, focussed on widows, a highly marginalised group across South Asia (Kermode et al, Devine et al) and also note that multiple group interventions focused on the needs of women with mental health problems and socio-economically disadvantaged (also a key group facing intersectional disadvantage as well as social exclusion). However we agree that we could not provide insights on how psychosocial support groups work for other groups such as those excluded by caste or LGBTQI status. We have recognised this shortcoming in the methodological limitations* and w*e have added the following statement.*

*We note however that the studies in this review do not address the relevance and acceptability of psychosocial group interventions for a number of socially excluded groups, such as people who identify as Dalit, disabled or Lesbian, Gay, Bisexual, Transgender, Intersex (LGBTI) and mentioned this in the limitations section.*

Several points in the discussion veer beyond the remit of their review. Can be potentially reduced in length keeping to the findings in the current review.

*Thanks – we have extensively revised the discussion to focus better on findings in the current review and it is substantially shortened.*

Minor/discretionary

- On p.3, see phrase “..which seek provide an explanatory analysis”. Check grammar.

*Thanks – corrected.*

- PRISMA diagram (fig 1) has a remnant of grammar auto-correct while taking a screenshot (probably) which appears over the word “removed”.

*Thanks – corrected.*

- The list of countries covered in the review appears too often (3 times?)

*Thanks – corrected*

- On p.13, both “collective action” and collective strength are indicated as mechanism 4 at diff points.

*Thanks – corrected*

- On p.13, need not capitalise O in Figure “One”

*Thanks – corrected*

Reviewer #2: Thank you for inviting me to peer review the manuscript ‘Addressing mental ill-health through psycho-social groups in South Asia – a scoping review using a realist lens’. I have concerns about the methodological rigor and/or reporting of this work. Please find below a few comments to consider:

Abstract
(1) Form authors’ descriptions, it is not clear (i) which databases (including platforms) were searched (ii) for what type of literature (iii) published in what languages. In general, systematic search methodology for scoping reviews should be explicit, transparent, and reproducible.

*Response – All these details are now provided in the methods section in the main paper and we have clarified details as permitted by space in the abstract.*

(2) What is the justification for the publication date restriction, i.e., 2007 onwards?

*Response – we have explained this in Methods section (date of the launch of the first Lancet series on global mental health)*

(3) Not clear from the authors’ descriptions if grey literature was searched, please expand.

*Response – We have explained that we sought grey literature but the majority did not meet basic quality criteria so it was not utilised.*

(4) Did the authors develop a protocol for this review? If yes, was it registered with any of the platforms or published? If not, please explain.

*Response – Yes – a protocol was published and this is now detailed in the methods*

(5) Did the authors consider a critical appraisal of the literature given they are looking at intervention research? If yes, what tool did they use?

*Response – We have explained that we performed a simple critical appraisal and the bespoke tool we used for this – given the highly heterogeneous and mixed methods of papers in this review.*

(6) Did the authors follow the PRISMA extension for scoping reviews?

*Yes – we have filled the Prisma scoping review checklist in the Supplementary files*

(7) It will be informative to report here the number and characteristics of participants the included interventions report on.

*Response – Table provided in supplementary material provided provides an overview of study participants and group characteristics.*

Methods and materials
(8) I would suggest rewriting the methods sections as it reads like an outline of the protocol.

*Response – Thank you for noting this. We have now reorganised the section and start from a definition of key concepts.*

(9) Page 5- Design/Research type - I am not clear why study protocols were included?

*Response – Thank you for the good question. In general, for each intervention, we included as much published information as possible. Since interventions may sometimes be described in greater detail in study protocols, we also consulted study protocols for more information about what was done. We have clarified this in the paper. several of them positied mechanisms of how they intervention might work, which were details not provided in subsequent papers. As this was a key focus of our study we wanted to include this detail.*

(10) The authors should make it clear to the reader what the inclusion and exclusion criteria were. Did the authors exclude commentaries, books, abstracts, and theoretical studies, opinion pieces, newsletters, magazines, newspapers?

*Response We only included studies that described and evaluated interventions. This specifically excludes the categories you highlighted. Exclusion criteria are supplied in the supplementary material.*

(11) What is the definition of adults in this work? I can see from Supplementary file A that interventions targeting children (14 and 15 years olds) were included, please explain.

*Response – Thank you for noting this. Some of the interventions we studied targeted both adults and young people. We excluded interventions that only targeted young people.Clarified age range on p5.*

(12) Systematic search methodology for scoping reviews should be explicit, transparent, and reproducible. Further, the authors should provide a complete search for at least one database (e.g., MEDLINE) so it is clear for the reader what type of MeSH terms and keywords were used, which terms were exploded, restrictions applied and the number of hits retrieved for each search string. Please add a full search for MEDLINE.

*Response –* A full MEDLINE search has been added to the supplementary material.

(13) Could the authors explain Table 1?

(i) Where these items are coming from? Why the authors did not use a validated quality assessment tool that would account for all types of studies?
(ii) How the results of the quality assessment exercise have been used in findings.
(iii) What do the authors mean by intervention studies? Why intervention studies are described separately from quantitative studies. RCTs are interventions that report quantitative evidence.

*Response –Thank you for this question. We include all studies that describe interventions, regardless of whether these interventions are evaluated qualitatively or quantitatively. For example, the realist evaluation paradigm relies heavily on qualitative data.*

*We have responded to these questions with the following text added:*

*Typically, scoping reviews do not include appraisals of the publications reviewed. We decided to report basic appraisal information to inform the planning of further dedicated qualitative and quantitative syntheses. We created a custom tool with three main parts: Quality and depth of the intervention description; Quality of any quantitative findings (informed by the CONSORT reporting guidelines); Quality of any qualitative findings (informed by the SRQR reporting guidelines).*

*The tool is summarised in Table 1. It was applied on the level of the intervention, not on the level of individual papers, since we recognise that different analyses are described in varying levels of detail in different publications.*

(14) I am not clear why the authors report on Ethical considerations for the scoping review, please explain.

*Response – we have clarified the ethics permission was sought to address inclusion of data from the two reference panels*

(15) I would urge the authors to conform to the PRISMA extension for scoping review guidelines.

*Response – we have filled the PRISMA extension checklist for scoping review guidelines and attached in Supplementary files.*

Results/Discussion
(16) From the authors’ descriptions, I am not clear what type of evidence was extracted from what type of studies.

Thank you – we have revised the Methods and materials section and clarified what data was extracted with this phrasing:

We documented how the outcomes of the interventions were measured, which scales were used, and how they were reported qualitatively as well as quantitatively. We paid particular attention to potential mechanisms reported in qualitative findings or in the discussions of included studies.

We extracted study characteristics that were relevant to the context of the intervention, the type of psycho-social group mental-health intervention, information about the implementation of these groups and the composition of these groups, barriers to participation, outcomes linked to psychosocial groups, and potential mechanisms for these outcomes. Quantitative study characteristics and outcomes were summarised descriptively. Qualitative outcomes were analysed using thematic analysis, as suggested by Levac et al. (44).

(17) In the peer-review PDF, I cannot see Table 2 but I have access to 2 PRISMA diagrams. I am therefore unable to evaluate if the data and analyses support the results and discussion.

*Response – we have now included all the Tables within the main document.*

(18) What about the effectiveness of interventions? I cannot see any synthesis and further discussion around effect sizes coming from RCTs, please explain.

*Response – Evaluating effectiveness wasn’t the primary goal of this study and the highly disparate study design and outcomes and measures did not lend themselves to further analysis of effect sizes.*

(19) I am not clear from the title what Table 3 describes. Are those measures/validated tools used for measuring mental health in interventions? If yes, why social factors are included as an outcome, please expand on this.

*Response – we have updated the title of Table 3 to read: Table 3. Variables and scales used for social and health outcomes in this review. As the premise of this review recognises that many social determinants of health are critical in determining mental health outcomes, we have included a wide range of outcomes which include social factors. The Introduction explains this stance.*

Response.
(20) In the peer-review PDF, I cannot see Table 4. I am therefore unable to evaluate if the data and analyses support the results and discussion.

Response - we *have now included all the Tables within the main document.*

(21) I would suggest the review findings are described separately from the engagement work with panelists and other stakeholders.

*Response – The findings from reference panels are at the end of each section and clearly identified as such now.*

(22) The manuscript will benefit from revision for typographical errors.

*Response – Thanks – we have revised and corrected typographical errors.*

6. PLOS authors have the option to publish the peer review history of their article ([what does this mean?](https://journals.plos.org/globalpublichealth/s/editorial-and-peer-review-process#loc-peer-review-history)). If published, this will include your full peer review and any attached files.


**Do you want your identity to be public for this peer review?** If you choose “no”, your identity will remain anonymous but your review may still be made public.


For information about this choice, including consent withdrawal, please see our [Privacy Policy](https://www.plos.org/privacy-policy).

Reviewer #1: **Yes:** Prashanth N Srinivas

Reviewer #2: No
