# Supplementary material for: Improving mental ill-health with psycho-social group interventions in South Asia–A scoping review using a realist lens
Source: PLOS Glob Public Health. 2023 Aug 28;3(8):e0001736. doi: 10.1371/journal.pgph.0001736 (PMC10461838; doi:10.1371/journal.pgph.0001736)
Supplement: Attachment — Submitted filename: Response to reviewers June 2023 F.docx [file pgph.0001736.s005.docx]

# Response to editors

Define the term - *psychosocial group intervention* at the beginning of the article,

**Response - Thanks – we have added this sentence to the introduction:**

*Psycho-social group interventions are defined in this study as structured cognitive, behavioural and social interventions intended to improve mental health implemented among a group of people who meet together on multiple occasions(1, 2).*

Remove information about the exclusion criteria from the supplementary material to the Methods section,

**Response - Thank you – we have added this sentence to the methods:**

*We excluded interventions that did not describe a group intervention or where groups were members of the same family or larger than 30 people in size, that were not set in South Asia, that were mainly delivered by health care professionals or were mainly biomedical, that had a primary focus on training community health workers, that did not focus on improving psychological wellbeing or that gave insufficient detail of the intervention.*

Elaborate the listed limitations and strengths, explaining why they are strengths or limitations,

**Response - Thank you, we have re-written this whole section, giving greater detail about how the described strengths and limitations impact the utility of the findings.**

Update the references to the latest Global Burden of Disease Study,

**Response – thanks – we have updated this reference**

Explain all the points mentioned by the Reviewer 3,

**Response - Thank you – we have responded to each point in the text below**

Review the text for typos and errors, again.

**Response – thank you, we have responded to each point in the text below.**

# Responses to reviewers

Reviewer 3

Methods
• Confused by connection of search strategy to research questions - Research questions focus on “psycho-social group” interventions but “psycho-social group” and related terms, e.g., “peer support group” were not included in search
•	*Response – Thanks for this comment. We had provided only some examples of search terms in the Methods and also one sample Search strategy in Methods meaning we included additional terms in our search. We have now edited and described our Search Strategy section of the paper as follows (paragraph 2):*

……. The search strategies consisted of three sets of terms, one set consisting of the names of each of the eight SAARC countries, one set for mental health, which included terms such as “mental health”, “psycho-social”, “mental disease”, “mental wellbeing”, “mental stress”, “social support”, and one set for the delivery mechanism, which included terms such as “community care”, “community health”, “task shifting”, “mental health care”, and “mental health service”, “group”. Within each of the three sets, terms were joined by „or”. The search terms were chosen to ensure maximum coverage of different approaches, given that such groups are described using many different terms. In particular, we wanted to ensure coverage of interventions that follow psychosocial principles, but do not describe themselves as such.  We developed the search terms based on a mini review (3). The three sets themselves were joined by “and”. We searched for papers that contained the terms in their title, abstract, or keywords.

“Exclusion criteria are supplied in the supplementary material.” – should be included in main body of paper in Methods section

Response – Thank you – we have added this sentence into the Methods section.

*We excluded interventions that did not describe a group intervention or where groups were members of the same family or larger than 30 people in size, that were not set in South Asia, that were mainly delivered by health care professionals or were mainly biomedical, that had a primary focus on training community health workers, that did not focus on improving psychological wellbeing or that gave insufficient detail of the intervention.*

• Further clarify sentence “It was applied on the level of the intervention, not on the level of individual papers, since we recognise that different analyses are described in varying levels of detail in different publications.”

Response – thanks for this query – we have further clarified this statement as follows:

*We did this because different analyses from the same intervention provided different levels of detail, and a focus on an intervention (which be published with multiple outputs) gave us the most complete information to assess the quality of an intervention.*

• Why only meetings with experts in 2 countries when multiple other countries included in South Asia? Why Practitioner and Lived Experience panels only in India?

Response – thanks for this comment. We had noted this as a methodological limitation and have further expanded this now as follows:

Third, while we reviewed literature on SAARC countries, our stakeholder engagement was limited to India due to a limited budget which precluded the costs of facilitation and travel to conduct reference panels in other South Asian countries. Contributions from researchers in other SAARC contexts may offer further insight into the commonalities and differences that facilitate the benefits of psychosocial interventions within local communities.

Discussion
• The issue of measures (p. 25) could be elaborated on, i.e., what is the impact of non-locally developed measures on study findings?

Response- thankyou for this comment. We have added the following text into the Mehtods section

*Psychometric measures can be less valid or accurate when they are used in a different setting or country to the place where they were originally developed. In a new setting they may fail to describe or represent the features of mental health or sickness which are determined culturally, socially and politically (4). There is an ‘ongoing need for in the conceptualization and measurement of culture- specific psychopathology and in developing culturally responsive interventions’ (5). Outcomes were not easily comparable because of the range of interventions, measures, and types of groups, as well as the diverse nature of the study contexts. Table 3 summarises quantitative measures used in the papers reviewed.*

And we have added the following to the Strengths and limitations section

Fifth, recognising that the majority of psychometric measures used in this study were developed outside of South Asia, the measures may be less accurate and relevant for a South Asian setting compared to the place where they were first developed.

• Methodological Considerations sub-heading could be revised to Strengths and Limitations
- 1st para should be included in methods section; more on study motivation and organization responsible should be included in Intro

**Response – thanks for this comment – we have moved this paragraph to the start of methods and elaborated further as pasted below:**

This study was motivated by a question posed by practitioners who had noted the effectiveness of group platforms to improve mental health and social inclusion in a youth resilience intervention, Benefits were noted for participants almost regardless of the content of an intervention(6). Practitioners (KM, PP and VS) were working with Burans, a non-profit community health initiative based in North India and administered by Herbertpur Christian Hospital, a member of the Emmanuel Hospital Association (7). Two published studies describe the organisational practices and approaches of Burans, and describe the value of attentive and innovative community mental health practice by local non-profit providers(8, 9). A research question with a local pose and gaze means it is more likely to be relevant and to lead to practical applications (10). The search strategy and analysis were refined iteratively, in engagement with practitioners which included representatives from both southern and northern India and also included people who are experts by experience. Findings were triangulated with reference panels of local experts, who were able to engage with findings and increase their relevance and contextual validity.

- Limitations and strengths should be elaborated on – rather than just listing them, explain why they are strengths or limitations

**Response - Thank you, we have re-written this whole section, giving greater detail about how the described strengths and limitations impact the utility of the findings.**

- Some editorial revisions needed in this section (typos, errors)

**Response – thank you, we have proof-read and edited the whole paper again.**

Conclusion
• “This is the first review globally to exclusively examine the contribution of psycho-social groups as a mental health intervention.”
- This isn’t true – may be true that it is first review in South Asian region?

Response – thanks, we have searched with a range of search terms and have not found any other review in other settings – we are happy to cite such a review if we are informed of the published articles. However we have changed the text to read:

This is the first review to our knowledge to examine the contribution of psycho-social groups as a mental health intervention in low- and middle-income countries.

Define “psycho-social group intervention” at start of paper

– we have included a definition in the second paragraph of the introduction that reads

*Psycho-social group interventions are defined in this study as structured cognitive, behavioural and social interventions intended to improve mental health implemented among a group of people who meet together on multiple occasions(1, 2).*

References
- Update to latest GBD Study (2019)

Response – thanks – we have updated our references accordingly.

## References cited in the response to reviewers/ editors

1.	Galappatti A. What is a psychosocial intervention? Mapping the field in Sri Lanka. The International Journal of Mental Health, Psychosocial Work and Counselling in Areas of Armed Conflict. 2003;1(2):3-17.

2.	van der Heijden I, Abrahams N, Sinclair D. Psychosocial group interventions to improve psychological well‐being in adults living with HIV. Cochrane Database of Systematic Reviews. 2017(3).

3.	Wolters M, Jain S, Fraser R, Macbeth A, Mathias K. Mini-Reviews For Iterative Scoping Review Protocol Development. Edinburgh, United Kingdom: University of Edinburgh; 2019.

4.	Kirmayer LJ, Young A. Culture and context in the evolutionary concept of mental disorder. J Abnorm Psychol. 1999;108(3):446-52.

5.	Hall GCN, Ibaraki AY, Huang ER, Marti CN, Stice E. A meta-analysis of cultural adaptations of psychological interventions. Behav Ther. 2016;47(6):993-1014.

6.	Mathias K, Singh P, Butcher N, Grills N, Srinivasan V, Kermode M. Promoting social inclusion for young people affected by psycho-social disability in India – a realist evaluation of a pilot intervention. Global Public Health. 2019;1744-1706.

7.	Burans. Burans website Dehradun district, Uttarakhand, North India: Herbertpur Christian Hospital; 2023 [Available from: www.burans.org.

8.	Srinivasan V, Jain S, Kwon W, Bayetti C, Cherian AV, Mathias K. What innovative practices and processes are used to deliver psychosocial care in India? A qualitative case study of three non-profit community mental health organisations. SSM-Mental Health. 2023:100220.

9.	Mathias K, Mathias J, Goicolea I, Kermode M. Strengthening community mental health competence – a realist informed case study from Dehradun, North India. Health & Social Care in the Community. 2018;26(1):e-179-90.

10.	Abimbola S. The uses of knowledge in global health. BMJ Specialist Journals; 2021.
